# Supplementary material for: An open-source framework for large-scale, flexible evaluation of biomedical text mining systems
Source: J Biomed Discov Collab. 2008 Jan 29;3:1. doi: 10.1186/1747-5333-3-1 (PMC2276192; doi:10.1186/1747-5333-3-1)
Supplement: Additional file 1 — Evaluation platform source code. The additional file contains the software evaluation framework discussed in this paper. The most current release can be downloaded from SourceForge.net [1]. See the accompanying README file for instructions on installation and use. Also included in the distribution is a collection of UIMA wrappers for some commonly used BioNLP tools and annotated corpora. [file 1747-5333-3-1-S1.zip › bionlp_uima_repository-1.0.1/docs/api/src-html/edu/uchsc/ccp/uima/ae/util/filter/AnnotationSetFilter_AE.html]

```
001    package edu.uchsc.ccp.uima.ae.util.filter;
002    
003    import java.util.ArrayList;
004    import java.util.Iterator;
005    import java.util.List;
006    
007    import org.apache.uima.analysis_engine.ResultSpecification;
008    import org.apache.uima.analysis_engine.annotator.AnnotatorConfigurationException;
009    import org.apache.uima.analysis_engine.annotator.AnnotatorContext;
010    import org.apache.uima.analysis_engine.annotator.AnnotatorContextException;
011    import org.apache.uima.analysis_engine.annotator.AnnotatorInitializationException;
012    import org.apache.uima.analysis_engine.annotator.AnnotatorProcessException;
013    import org.apache.uima.analysis_engine.annotator.JTextAnnotator_ImplBase;
014    import org.apache.uima.jcas.JCas;
015    import org.apache.uima.jcas.cas.FSArray;
016    
017    import edu.uchsc.ccp.uima.annotation.CCPAnnotationSet;
018    import edu.uchsc.ccp.uima.annotation.CCPTextAnnotation;
019    
020    /**
021     * removes all annotations not created by a particular annotator
022     * 
023     * @author williamb
024     * 
025     */
026    public class AnnotationSetFilter_AE extends JTextAnnotator_ImplBase {
027    
028        public static final String PARAM_ANNOTATIONSET_IDS_TO_KEEP_LIST = "AnnotationSetIDsToKeepList";
029    
030        private List<Integer> annotationSetIDsToKeep;
031    
032        @Override
033        public void initialize(AnnotatorContext ac) throws AnnotatorInitializationException, AnnotatorConfigurationException {
034            /* read in input parameters, and initialize a list of annotator ids to keep */
035            try {
036                Integer[] annotatorIDs = (Integer[]) ac.getConfigParameterValue(PARAM_ANNOTATIONSET_IDS_TO_KEEP_LIST);
037                annotationSetIDsToKeep = new ArrayList<Integer>();
038                for (Integer id : annotatorIDs) {
039                    annotationSetIDsToKeep.add(id);
040                }
041            } catch (AnnotatorContextException e) {
042                e.printStackTrace();
043            }
044            super.initialize(ac);
045        }
046    
047        /**
048         * cycle through all annotations and set the annotation set
049         */
050        public void process(JCas jcas, ResultSpecification arg1) throws AnnotatorProcessException {
051    
052            List<CCPTextAnnotation> annotationsToRemove = new ArrayList<CCPTextAnnotation>();
053    
054            Iterator annotIter = jcas.getJFSIndexRepository().getAnnotationIndex(CCPTextAnnotation.type).iterator();
055    
056            while (annotIter.hasNext()) {
057                Object possibleAnnot = annotIter.next();
058                if (possibleAnnot instanceof CCPTextAnnotation) {
059                    CCPTextAnnotation ccpTA = (CCPTextAnnotation) possibleAnnot;
060                    FSArray ccpAnnotationSets = ccpTA.getAnnotationSets();
061                    boolean keep = false;
062                    for (int i = 0; i < ccpAnnotationSets.size(); i++) {
063                        CCPAnnotationSet annotationSet = (CCPAnnotationSet) ccpAnnotationSets.get(i);
064                        Integer annotationSetID = new Integer(annotationSet.getAnnotationSetID());
065                        if (annotationSetIDsToKeep.contains(annotationSetID)) {
066                            keep = true;
067                        }
068                    }
069                    if (!keep) {
070                        annotationsToRemove.add(ccpTA);
071                    }
072                } else {
073                    System.err.println("WARNING -- AnnotationSetFilter_AE: CCPTextAnnotation expected but instead got "
074                            + possibleAnnot.getClass().getName());
075                }
076            }
077    
078            /* now remove annotations that had annotators not in the annotatorIDsToKeep list */
079            for (CCPTextAnnotation ccpTA : annotationsToRemove) {
080                ccpTA.removeFromIndexes();
081                ccpTA = null;
082            }
083    
084        }
085    
086    }
```
